# Editorial: Exploring and Engineering Plant Specialized Metabolism: Latest Advances and New Horizons

**DOI:** 10.3389/fpls.2021.783465

**Published:** 2021-10-28

**Authors:** Jakob Franke, Yang Zhang, Thu-Thuy T. Dang

**Affiliations:** ^1^Institute of Botany, Leibniz University Hannover, Hanover, Germany; ^2^Centre of Biomolecular Drug Research, Leibniz University Hannover, Hanover, Germany; ^3^Key Laboratory of Bio-resource and Eco-environment of Ministry of Education, College of Life Sciences, Sichuan University, Chengdu, China; ^4^Department of Chemistry, Irving K. Barber Faculty of Science, The University of British Columbia, Kelowna, BC, Canada

**Keywords:** genomics, transcriptomics, plant biochemistry, transcription factors, metabolic engineering

Plants use specialized metabolic pathways to produce over 200,000 small molecules which often have potent biological activities. Many of these compounds have medicinal, nutritional or other applications. However, the natural supply from the producing plants is often strongly limited, for example because plants produce insufficient quantities or do not produce biomass fast enough. There is therefore an urgent need to improve our understanding of plant specialized metabolism, and to come up with strategies to engineer the underlying pathways. This is especially true in the light of climate change and the mandate to transition to a biobased economy. For that reason, this Research Topic aims to collate recent developments in the area of plant specialized metabolism, with a special focus on cutting-edge methods to explore as well as engineer biosynthetic pathways.

Probably the most important technical progress of the last decade has been achieved in the field of sequencing platforms. Nowadays, obtaining transcriptome and even genome data of non-model plant species is a realistic option even for smaller labs. For example, high-quality genome assemblies up to the chromosome level have been reported recently for medicinal plants such as *Senna tora* (Kang et al., 2020), *Camptotheca acuminata* (Zhao et al., 2017; Kang et al., 2021), *Ophiorrhiza pumila* (Rai et al., 2021), *Papaver somniferum* (opium poppy; Guo et al., 2018; Li et al., 2020), and *Taxus chinensis* var. *mairei* (Xiong et al., 2021) as well as for culinary herbs from the mint family (Lamiaceae; Bornowski et al., 2020; Lichman et al., 2020). This advance opens up numerous avenues to gain a better understanding of plant specialized metabolism on a transcriptome- or genome-wide level.

The importance of sequencing data for investigating the biochemistry of understudied plant species is underlined very well by the publications in this Research Topic: For example, Yamada et al. performed a genome-wide profiling of WRKY transcription factor genes in California poppy (*Eschscholzia californica*) to study their effect on benzylisoquinoline alkaloid biosynthesis. A similar approach was used by Cao et al. with a focus on MYB transcription factor genes in Chinese Bayberry (*Morella rubra*) to investigate flavonoid metabolism. Li et al. used genome data from red sage (*Salvia miltiorrhiza*) to identify TIFY transcription factors involved in regulation of specialized metabolism. Lastly, Zhang et al. combined multiple omics techniques to gain a better understanding of how lipid and fatty acid synthesis is regulated in sesame seeds (*Sesamum indicum*).

While all of these studies demonstrate well how current omics techniques can be applied to understand regulatory circuits of already known plant metabolic pathways, there remains a much larger number of pathways that have yet to be elucidated. The key challenge here is to identify the genes and enzymes involved in these pathways, which is often a slow and tedious process and requires an efficient bioinformatic and biochemical pipeline. However, several break-through publications in the last years demonstrate that plant pathway elucidation is now becoming increasingly feasible (Caputi et al., 2018; Dang et al., 2018; Christ et al., 2019; Hodgson et al., 2019; Pluskal et al., 2019; Nett et al., 2020). In the course of these and numerous other projects, many unusual and powerful enzymes have been discovered, which are also attractive from a biocatalysis perspective. A particularly important class of enzymes are cytochromes P450, whose broad repertoire of catalytical function was reviewed by Nguyen and Dang. Again, analyzing genome and transcriptome data *via* state-of-the-art bioinformatics approaches has been key to discovering novel biosynthetic genes and enzymes from plants. Plant biosynthetic gene clusters are now reported more and more frequently, as plant genomic data can be obtained more readily. An overview over currently known plant biosynthetic gene clusters is provided by Bharadwaj et al..

Understanding how plant metabolic pathways work and are regulated is key to engineer them successfully. A strategy that is commonly used is to transfer these pathways into baker's yeast (*Saccharomyces cerevisiae*) as a versatile and easy-to-handle eukaryotic host system. As reviewed by Utomo et al., this success is particularly based on CRISPR/Cas9-based techniques for multiplex genome editing. However, not only microorganisms are attractive hosts for pathway engineering. Thanks to advancements in the fields of genome editing and plant biochemistry, original producer plants are now often engineered rationally as well. Examples from engineering terpenoid metabolism in glandular trichomes of Lamiaceae plants are reviewed by Mahmoud et al. How a better understanding of plant metabolism can translate into a relevant application is also demonstrated by the article of Koudounas et al.; in their work, they successfully silenced a gene involved in secoiridoid biosynthesis in olives (*Olea europaea*), which might be valuable to improve the effects of olive oil on human health.

As demonstrated by this Research Topic, the impact of modern sequencing techniques, bioinformatics analysis platforms, state-of-the-art biochemical approaches and new genetic engineering techniques to the field of plant specialized metabolism has been tremendous. This has enabled various biochemical discoveries and engineering applications, which would not have been possible only a few years ago. We are looking forward to seeing further progress in understanding plant specialized metabolism and additional real-world applications of pathway engineering in the years to come.

## Author Contributions

All authors listed have made a substantial, direct and intellectual contribution to the work, and approved it for publication.

## Funding

YZ was supported by the Institutional Research Fund of Sichuan University (2020SCUNL106) and the Fundamental Research Funds for the Central Universities (SCU2021D006).

## Conflict of Interest

The authors declare that the research was conducted in the absence of any commercial or financial relationships that could be construed as a potential conflict of interest.

## Publisher's Note

All claims expressed in this article are solely those of the authors and do not necessarily represent those of their affiliated organizations, or those of the publisher, the editors and the reviewers. Any product that may be evaluated in this article, or claim that may be made by its manufacturer, is not guaranteed or endorsed by the publisher.
